# Supplementary material for: DMRforPairs: identifying Differentially Methylated Regions between unique samples using array based methylation profiles
Source: BMC Bioinformatics. 2014 May 15;15:141. doi: 10.1186/1471-2105-15-141 (PMC4046028; doi:10.1186/1471-2105-15-141)
Supplement: Additional file 2 — DMRforPairs output for the comparison of A431-MCF7 and NA17018-NA17105. Please start from the HTML files in each folder. Available via the BMC Bioinformatics website. [file 1471-2105-15-141-S2.zip › 1394847754114233_MOESM2_ESM/A431_MCF7/figures/4778.pdf]

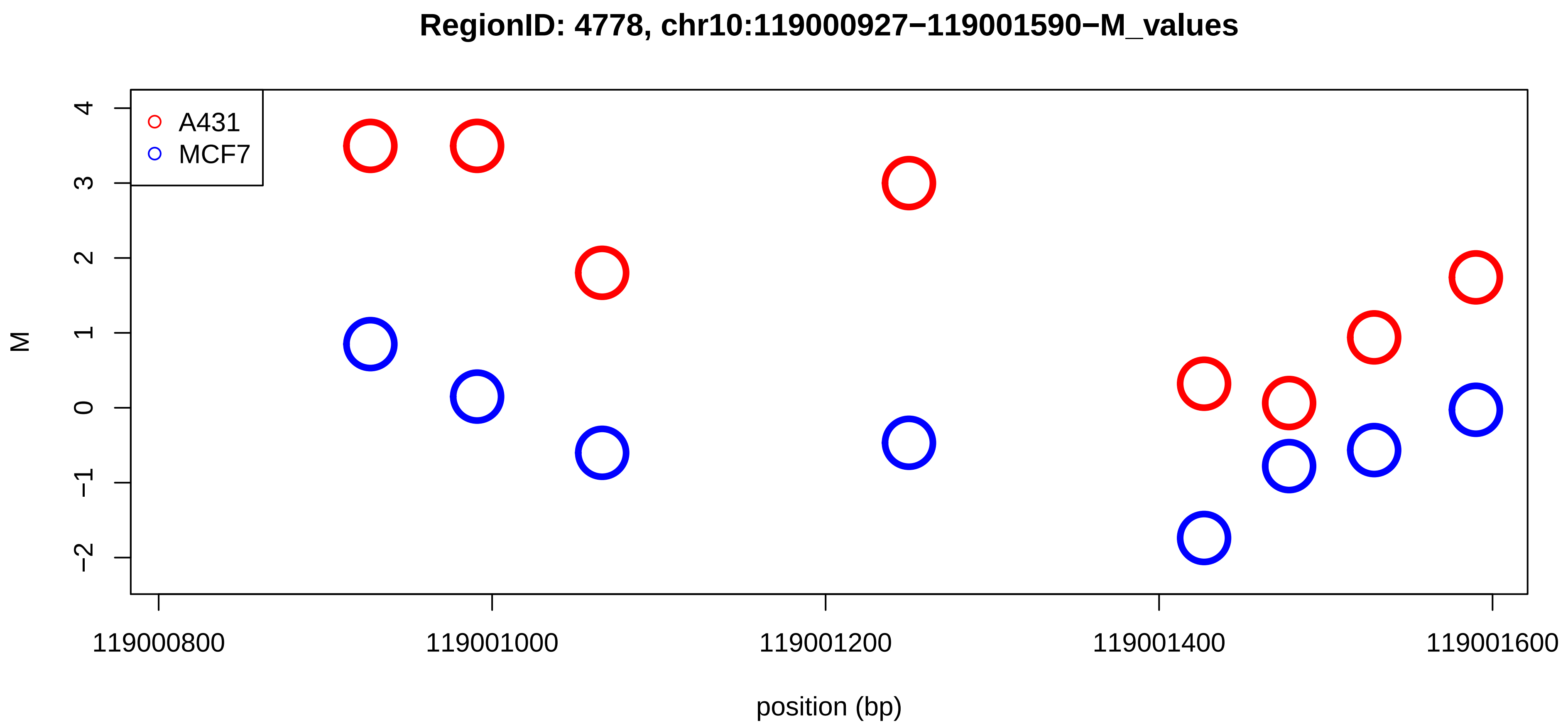
RegionID: 4778, chr10:119000927−119001590−M_values
A431
MCF7
M
4
3
2
1
0
−1
−2
119000800
119001000
119001200
119001400
119001600
position (bp)

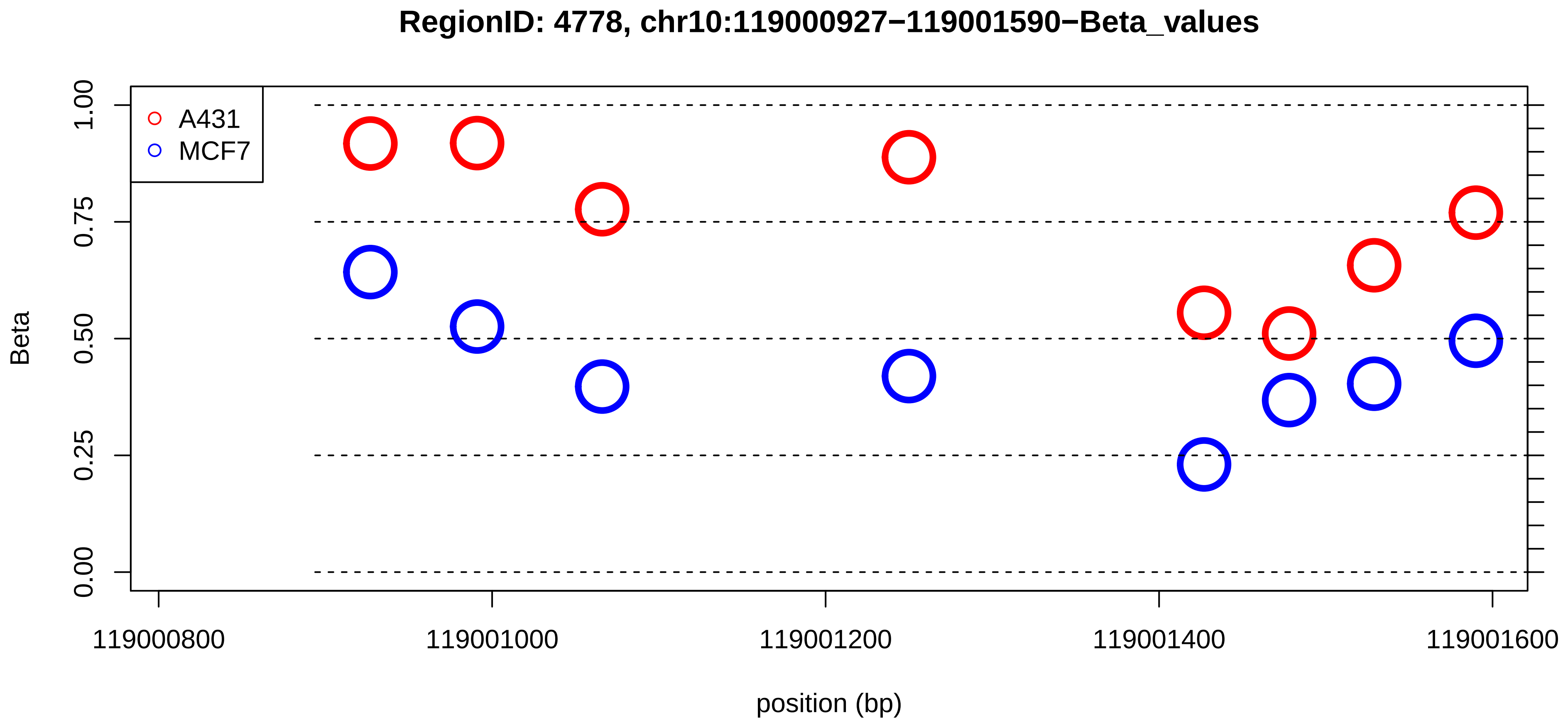
RegionID: 4778, chr10:119000927−119001590−Beta_values
A431
MCF7
Beta
1.00
0.75
0.50
0.25
0.00
119000800
119001000
119001200
119001400
119001600
position (bp)

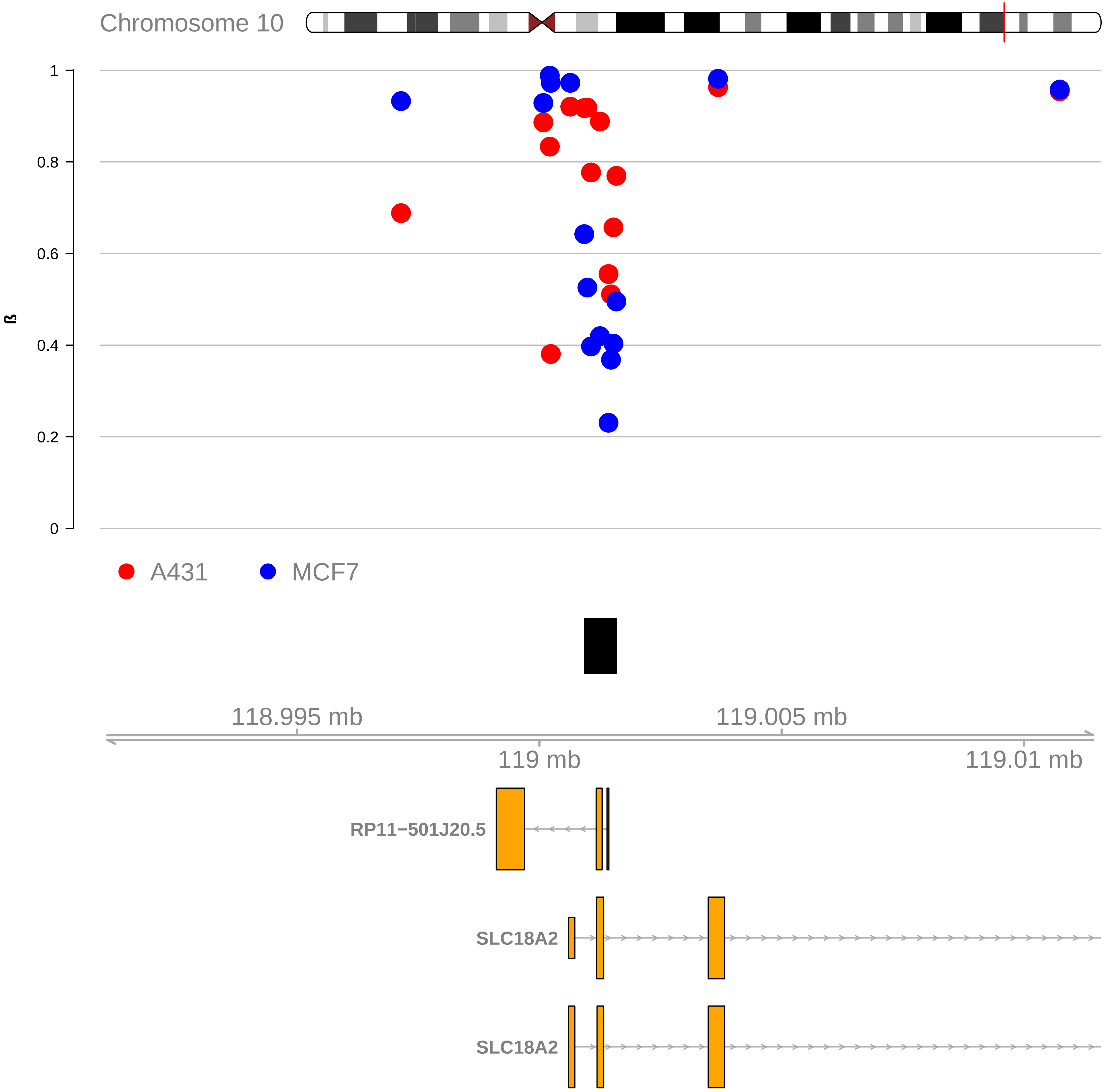
Chromosome 10
1
0.8
0.6
0.4
0.2
0
ß
A431
MCF7
118.995 mb
119.005 mb
119 mb
119.01 mb
RP11−501J20.5
SLC18A2
SLC18A2
